# High rate of bleeding and arterial thrombosis in COVID-19: Saudi multicenter study

**DOI:** 10.1186/s12959-021-00265-y

**Published:** 2021-03-03

**Authors:** Abdulrahman Al Raizah, Ahmed Al Askar, Naila Shaheen, Khalid Aldosari, Mohamed Alnahdi, Musumadi Luhanga, Turki Alshuaibi, Wail Bajhmoum, Khaled Alharbi, Ghaida Alsahari, Hadeel Algahtani, Eunice Alrayes, Abdulrahim Basendwah, Alia Abotaleb, Mosaad Almegren

**Affiliations:** 1 Division of Adult Hematology, Department of Oncology, King Abdulaziz Medical City, Ministry of National Guard Health Affairs, PO Box. 22490, 11426 Riyadh, Saudi Arabia; 2 King Abdullah International Medical Research Center, Saudi Society for Bone Marrow Transplant, Ministry of National Guard Health Affairs, Riyadh, Saudi Arabia; 3 King Saud bin Abdulaziz University of Health Sciences, Riyadh, Saudi Arabia; 4 Department of Biostatistics and Bioinformatics, King Abdullah International Medical Research Center, Riyadh, Saudi Arabia; 5 Department of Medicine, King Fahd Hospital, Jeddah, Saudi Arabia; 6 Department of Medicine, Prince Mohammed Bin Abdulaziz Hospital, Riyadh, Saudi Arabia; 7 Oncology Division, Medicine Department, King Fahad Armed Forces Hospital, Jeddah, Mecca, Saudi Arabia; 8 Department of Medicine, College of Medicine, Imam Mohammad Ibn Saud Islamic University, Riyadh, Saudi Arabia

**Keywords:** SARS-CoV-2, COVID-19, Coronavirus, Bleeding, Thrombosis, Venous thromboembolism, Arterial thrombosis, Stroke, Saudi

## Abstract

**Background:**

Several observational studies have reported the rate of venous and arterial thrombotic events in patients infected with COVID-19, with conflicting results. The aim of this study was to estimate the rate of thrombotic and bleeding events in hospitalized patients diagnosed with Coronavirus disease 2019 (COVID-19).

**Methods:**

This was a multicenter study of 636 patients admitted between 20 March 2020 and 31 May 2020 with confirmed COVID-19 in four hospitals.

**Results:**

Over a median length of stay in the non-ICU group of 7 days and of 19 days in the ICU group, twelve patients were diagnosed with Venous thromboembolism (VTE) (1.8 %) (95 % CI, 1.1–3). The rate in the non-ICU group was 0.19 % (95 % CI, 0.04–0.84), and that in the ICU group was 10.3 % (95 % CI, 6.4–16.2). The overall rate of arterial event is 2.2 % (95 % CI, 1.4–3.3). The rates in the non-ICU and ICU groups were 0.94 % (95 % CI, 0.46–0.1.9) and 8.4 % (95 % CI, 5.0–14.0). The overall composite event rate was 2.9 % (95 % CI, 2.0–4.3). The composite event rates in the non-ICU and ICU groups were 0.94 % (95 % CI, 0.46–0.1.9) and 13.2 % (95 % CI, 8.7–19.5). The overall rate of bleeding is 1.7 % (95 % CI, 1.0–2.8). The bleeding rate in the non-ICU group was 0.19 % (95 % CI, 0.04–0.84), and that in the ICU group was 9.4 % (95 % CI, 5.7–15.1). The baseline D-dimer level was a significant risk factor for developing VTE (OR 1.31, 95 % CI, 1.08–1.57, *p* = 0.005) and composite events (OR 1.32, 95 % CI, 1.12–1.55, *p* = 0.0007).

**Conclusions:**

In this study, we found that the VTE rates in hospitalized patients with COVID-19 might not be higher than expected. In contrast to the risk of VTE, we found a high rate of arterial and bleeding complications in patients admitted to the ICU. An elevated D-dimer level at baseline could predict thrombotic complications in COVID-19 patients and may assist in the identification of these patients. Given the high rate of bleeding, the current study suggests that the intensification of anticoagulation therapy in COVID-19 patients beyond the standard of care be pursued with caution and would best be evaluated in a randomized controlled study.

## Background

Venous thromboembolism (VTE) is a leading cause of preventable hospital mortality [1]. Approximately 50 % of VTE events occurring outside of a hospital are due to recent hospitalization [2]. There are several risk factors for hospital acquired VTE, including acute illness, surgery, obesity, trauma, limited mobility, the presence of a central venous catheter, a history of thrombosis and old age [3]. The increased risk for VTE may persist for months after discharge [4]. It is estimated that 70 % of hospital-acquired VTE can be prevented through pharmacological or mechanical methods; however, less than 50 % of patients receive such preventive measures [5–7]. Coronavirus disease 2019 (COVID-19) is a global pandemic that has had a substantial impact on mortality, the health system and the economy [8, 9]. Several observational studies have reported the rate of venous and arterial thrombotic events in patients infected with COVID-19, with conflicting results. Early studies showed an increased risk of thrombosis in COVID-19 patients, especially in critically ill patients, with a crude cumulative composite outcome of venous and arterial events of 57 % [10–14] However, some studies reported a low rate of thrombotic events [15–18]. The aim of this multicenter study was to estimate the rate of thrombotic and bleeding events in hospitalized patients diagnosed with COVID-19 in Saudi Arabia.

## Methods

### Patients and data collection

We included consecutive patients aged ≥ 18 years admitted between 20 March 2020 and 31 May 2020 with confirmed COVID-19. This study was conducted in four different hospitals, community-based and academic teaching hospitals. A confirmed COVID-19 case was defined as a positive reverse-transcriptase polymerase chain reaction (RT-PCR) test by nasal or oropharyngeal swab. Patients were excluded if they were transferred in or out from one of these four hospital to another hospitals. Patients also excluded if they were admitted for less than 24 h. The patients were categorized as intensive care unit (ICU) patients or ward patients. ICU patients could be admitted to the ICU at any time during hospital admission. Data were collected retrospectively through a manual chart review from electronic and paper medical records from the first day of admission until discharge, death or the end of the data collection period (15 July 2020). The data were collected using a standardized form and included baseline characteristics, comorbidities, ICU admission, length of hospital stay, hospital discharge or death, bleeding and thrombotic events, dose and type of anticoagulation used, mechanical prophylaxis use and coagulation parameters. This study was approved by the Institutional Review Boards of all four hospitals. Due to the retrospective nature of this study, approval for written informed consent was waived.

### Outcomes

The primary outcome was the rate of VTE. The secondary outcomes were the rate of arterial events, the rate of composite events (venous and arterial) and the rate of bleeding. VTE included all symptomatic or incidentally diagnosed cases of pulmonary embolism (PE), deep vein thrombosis (DVT) and thrombosis in unusual sites (cerebral, mesenteric, portal, splenic, hepatic, and renal veins). All VTEs were confirmed radiographically by appropriate imaging. We also included PE that was not confirmed radiologically but highly suspected by the treating physician based on a combination of clinical signs, symptoms, cardiac enzymes, electrocardiogram findings, and/or echocardiogram monitoring findings. Screening for VTE in asymptomatic patients was not performed. If more than on type of VTE occurred in the same patient, it was considered one event. Arterial events included cerebrovascular accidents (CVAs), mesenteric ischemia, and limb ischemia and were confirmed by the appropriate imaging modality. Myocardial infarction (MI) was diagnosed based on the suspicion of the attending physician using clinical criteria as well as biomarker elevations or electrocardiographic changes. Composite events were defined as any VTE or arterial event. Bleeding events were classified as major and nonmajor based on the definition proposed by the International Society of Thrombosis and Haemostasis (ISTH). Major bleeding was defined as fatal bleeding; symptomatic bleeding in a critical organ, such as intracranial, intraocular, intraspinal, intra-articular, retroperitoneal, or pericardial bleeding; intramuscular bleeding with compartment syndrome; bleeding leading to a decrease in the hemoglobin level of 20 g/L or more; or the transfusion of 2 or more red blood cell units. All other bleeding episodes were considered nonmajor bleeding [19]. The baseline D-dimer level was defined as the first D-dimer level after admission. All D-dimer results are reported in fibrinogen equivalent units, with the normal level for D-dimer being less than 0.5 mcg/ml. Two centers used the INNOVANCE D-dimer assay (Siemens), and the other 2 centers used the STA-Liatest assay (Stago).

### Statistical analysis

Sex, nationality, history of cancer, history of thrombophilia, history of DVT, mechanical prophylaxis, enoxaparin dose, arterial thrombosis, location of the thrombus, lung infiltrate, and mortality are summarized as frequencies and percentages. The variables were compared in terms of the ICU and non-ICU groups using the chi-square/Fisher exact test. Data normality was determined with the Shapiro-Wilks test and is graphically displayed as a Q-Q plot. Age, cumulative comorbidities, length of hospital stay, and DVT duration were compared using the t test. The rates of VTE, bleeding, arterial and composite events are summarized as proportions, with the corresponding 95 % confidence intervals (CIs). The risk factors for developing VTE *(DVT/PE/other venous thromboembolism)* were identified using logistic regression. The independent variables age, sex, IMPROVE risk score, weight, baseline D-dimer level, baseline platelet count, and length of hospital stay were selected based on clinical judgment and univariate analyses. The results are reported as the odds ratios and the corresponding 95 % CIs and p-values.

Overall survival was compared between patients who had a composite event vs. those who had no composite event using the Kaplan-Meier method with the log-rank test. A p-value less than 0.05 was considered significant. Statistical analyses were performed using SAS version 9.4 (SAS Institute, Cary, NC, USA).

## Results

**Fig. 1:**
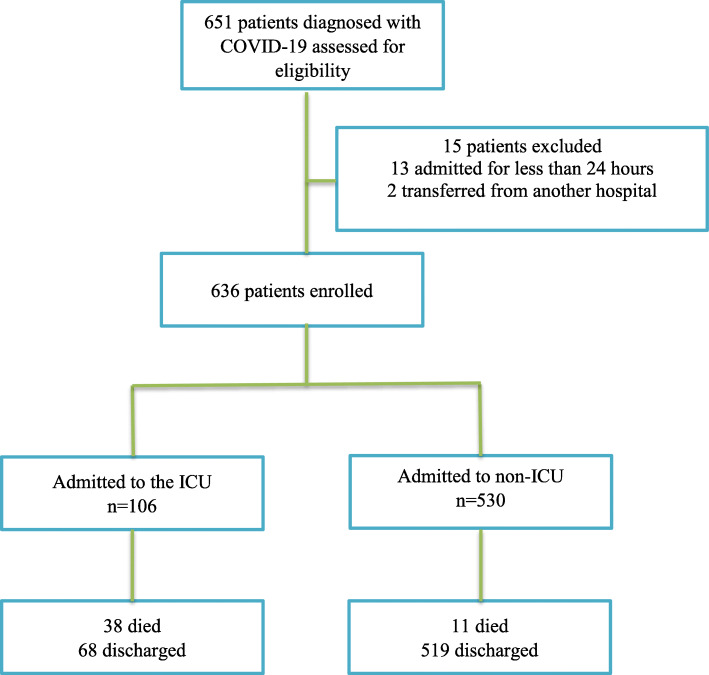
Flow diagram of the study population

In total, 651 patients diagnosed with COVID-19 were enrolled in the current study, with a final sample of 636 patients after exclusion (Fig. 1).

The demographic and baseline characteristics of the patients by severity group (ICU vs. non-ICU) are displayed in Table 1. The mean patient age was 49 ± 16 years, and the majority (71 %) were male. The mean BMI was 28 ± 6 kg/m^2^. One hundred and six patients (16.6 %) required ICU admission. The median length of hospitalization stay in the non-ICU group was 7 days, whereas that in the ICU group was 19 days. At the end of data collection (15 July 2020), 7.7 % (*n* = 49) of participants had died, and the rest were discharged. The majority of patients (90 %, *n* = 573) and more than 99 % of those in the ICU group received pharmacological prophylaxis according to local hospital practice. The most frequently prescribed regimen was enoxaparin (40 mg once daily) (59.2 %). Some patients were prescribed more than one regimen during admission. Full-dose anticoagulation was prescribed for 6.1 % of the sample due to a preexisting indication or empirically as part of COVID-19 management (Table 2).

**Table 1:** Demographics and Baseline Characteristics of the Population

	Overall *n* = 636	Non-ICU *n* = 530	ICU *n* = 106	*p*-value
Age (mean ± SD)	49 ± 16	47 ± 16	59 ± 14	< 0.0001
*Male n (%)*	456 (71.7)	371 (70)	85 (80.2)	0.033
*Female n (%)*	180 (28.3)	159 (30)	21 (19.8)	
Weight (mean ± SD)	78.1 ± 16.2	77.2 ± 15.1	82.8 ± 20.3	0.007
BMI (mean ± SD)	28.4 ± 6.0	28.1 ± 5.5	17.3 ± 7.8	0.075
^a^ *Any comorbidities n (%)*	303(47)	205(38)	98(92)	< 0.0001
^b^Active Cancer n (%)	12 (1.9)	9 (1.7)	3 (2.8)	0.432
History of Thrombophilia n (%)	3 (0.47)	1 (0.19)	2 (1.8)	0.073
History of DVT/PE n (%)	6 (0.94)	5 (0.95)	1 (0.94)	1.00

^a^Comorbidities: DM, hypertension, coronary artery disease, stroke, bronchial asthma, COPD, heart failure, and ESRD

^b^Active Cancer defined as patients on active treatment within last 6 months

**Table 2:** Treatment Received

Treatment	Overall *n* = 636 n (%)	Non-ICU *n* = 530 n (%)	ICU *n* = 106 n (%)	*p*-value
Mechanical prophylaxis	6 (0.94)	3 (0.57)	3 (2.8)	0.016
Pharmacological prophylaxis	573 (90.0)	468 (88.3)	105 (99.0)	0.0007
DOAC during admission	9 (1.4)	7 (1.3)	2 (1.8)	0.649
Antiplatelet	82 (12.9)	62 (11.7)	20 (19.2)	0.037
Enoxaparin Dose				
* 40 mg OD*	377 (59.2)	332 (62.6)	45 (42.4)	< 0.0001
* 40 mg BD*	35 (5.5)	18 (3.4)	17 (16.0)	
* 30 mg OD*	7 (1.1)	3 (0.57)	4 (3.7)	
* 30 mg BD*	2 (0.31)	0	2 (1.8)	
* Full dose*	28 (4.4)	20 (3.7)	8 (7.5)	
* Other dose*	24 (3.7)	17 (3.2)	7 (6.6)	
Unfractionated Heparin Dose				
* 5000 TID*	41 (6.4)	23 (4.3)	18 (16.9)	< 0.0001
* 5000 BID*	77 (12.1)	62 (11.7)	15 (14.1)	
* Full dose*	11 (1.7)	6 (1.1)	5 (4.7)	
* Other dose*	2 (0.32)	1 (0.19)	1 (0.94)	

### Thrombotic events

#### VTE

Twelve patients were diagnosed with VTE (1.8 %) (95 % CI, 1.1 − 3). The rate in the non-ICU group was 0.19 % (95 % CI, 0.04–0.84), and that in the ICU group was 10.3 % (95 % CI, 6.4–16.2). The VTE rate in the non-ICU group was 21 events (95 % CI, 11 − 38) per 10,000 person-days, and that in the ICU group was 83 events (95 % CI, 46–150) per 10,000 person-days. The cumulative incidence at 7, 14, and 21 days of VTE, arterial and bleeding outcomes are shown in Table 3.VTE was diagnosed a median of 13 days after admission. Six patients had PE, 3 had DVT (one was line related upper extremity), 2 had PE and DVT, and one had portal vein thrombosis. Regarding thromboprophylaxis, 2 patients were not on prophylaxis prior to the event, 3 were on enoxaparin (40 mg once daily), 3 were on UFH (5000 IU twice daily), 2 were on UFH (5000 IU TID), one was on enoxaparin (40 mg twice daily), and one was on fondaparinux (2.5 mg once daily). The type of all outcomes is displayed in Table 4.

**Table 3:** Cumulative Incidence at 7, 14 and 21 Days

Variables/day	All patients % (*95% CI)*	ICU % (*95% CI)*
*VTE*		
7	0.47 (0.16-1.3)	1.8 (0.51-6.6)
14	0.94 (0.43-2.0)	4.7 (2.0-10.5)
21	1.2 (0.63-2.4)	6.6 (3.2-13.0)
*Arterial*		
7	1.5 (0.85-2.8)	5.6 (2.6-11.8)
14	2.0 (1.1-3.4)	7.5 (3.8-14.1)
21	2.0 (1.1-3.4)	7.5 (3.8-14.1)
*Bleeding*		
7	0.16 (0.02-0.88)	0.94 (0.16-5.1)
14	0.94 (0.43-2.0)	4.7 (2.0-10.5)
21	1.2 (0.63-2.4)	6.6 (3.2-13.0)

**Table 4:** Outcome Data

Variable	Overall	Non-ICU	ICU	*P* value
VTE % (*95 % CI)*	1.8 (1.1–3)	0.19 (0.04–0.84)	10.3 (6.4–16.2)	< 0.0001
PE n	8	0	8	
DVT n	5	0	5	
* Proximal upper* n	1	0	1	
* Proximal lower* n	4	0	4	
Other VTE n	2	1	1	
Arterial % (*95 % CI)*	2.2 (1.4–3.3)	0.94 (0.46–1.9)	8.4 (5.0–14.0)	< 0.0001
* CVA* n	10	4	6	
* MI* n	4	1	3	
Bleeding *%* (*95 % CI)*	1.7 (1.0–2.8)	0.19 (0.04–0.84)	9.4 (5.7–15.1)	< 0.0001
* Major bleeding* n (%)	5	1	4	
* Other bleeding* n (%)	6	0	6	

#### Arterial events

Fourteen patients were diagnosed with an arterial event, with an overall rate of 2.2 % (95 % CI, 1.4–3.3). The rates in the non-ICU and ICU groups were 0.94 % (95 % CI, 0.46–0.1.9) and 8.4 % (95 % CI, 5.0–14.0). The arterial event rate was 71 events (95 % CI, 37–137) per 10,000 person-days. The arterial event rates in the ICU and non-ICU groups were 218 events (95 % CI, 104 − 457) per 10,000 person-days and 21 (95 % CI, 5 − 85) per 10,000 person-days.

#### Composite events

The overall composite event rate was 2.9 % (95 % CI, 2.0–4.3). The composite event rates in the non-ICU and ICU groups were 0.94 % (95 % CI, 0.46–0.1.9) and 13.2 % (95 % CI, 8.7–19.5). The composite event rate was 25 events (95 % CI, 15 − 42) per 10,000 person-days. The composite event rates in the ICU and non-ICU groups were 85 events (95 % CI, 48 − 151) per 10,000 person-days and 4 (95 % CI, 1 − 19) per 10,000 person-days.

#### Bleeding

Eleven patients developed bleeding, with an overall rate of 1.7 % (95 % CI, 1.0 − 2.8). The bleeding rate in the non-ICU group was 0.19 % (95 % CI, 0.04–0.84), and that in the ICU group was 9.4 % (95 % CI, 5.7–15.1). The overall bleeding rate was 22 events (95 % CI, 12 − 42) per 10,000 person-days, and the bleeding rate in the ICU group was 81 events (95 % CI, 42–157) per 10,000 person-days. Characteristic of bleeding are shown in Table 5.

**Table 5:** Bleeding Characteristic

Age	ICU	Site of bleeding	Severity	Anticoagulant at time of bleeding
45	Yes	Lung	Major	UFH 5000 IU TID
62	Yes	Lower GI	Major	Enoxaparin 40 mg once
62	Yes	Hematuria	Non major	Aspirin
61	Yes	Cutaneous	Non major	UFH 5000 IU TID
73	Yes	CNS	Major	Enoxaparin 40 mg once
64	Yes	Cutaneous	Non major	Enoxaparin 40 mg once
59	Yes	Cutaneous	Non major	Enoxaparin 40 mg once
62	No	Musculoskeletal	Major	Enoxaparin 40 mg once
31	Yes	CNS	Major	Enoxaparin 40 mg once
74	Yes	Cutaneous	Non major	Enoxaparin 40 mg BID
41	Yes	Lower GI	Non major	Full dose UFH

#### Risk factors for developing VTE/Composite events

From the available data, the only risk factor that predicted VTE and the composite outcome was baseline D-dimer levels (Tables 6 and 7). The baseline D-dimer level was a significant risk factor for developing VTE (OR 1.31, 95 % CI, 1.08–1.57, *p* = 0.005) and composite events (OR 1.32, 95 % CI, 1.12–1.55, *p* = 0.0007).

**Table 6:** Risk Factors of VTE

	*Odds Ratio*	*95 %CI*	*P-value*
Age	1.01	0.95–1.07	0.693
Gender (Females vs. *males*)	0.23	0.02–2.29	0.214
Improve Risk Score	3.36	0.68–16.48	0.134
Weight	1.01	0.98–1.06	0.177
Baseline D-Dimer	1.31	1.08–1.58	0.005
Baseline platelet	1.00	0.99-1.00	0.416
Length of hospital stay	1.01	0.96–1.07	0.547

The logistic regression model with firth correction is based on probability of having VTE

**Table 7:** Risk Factors of Composite Events

	*Odds Ratio*	*95 %CI*	*P-value*
Age	1.03	0.97–1.10	0.208
Gender (females vs. *males*)	1.09	0.25–4.75	0.905
Improve Risk Score	1.57	0.40–6.12	0.510
Weight	0.98	0.94–1.02	0.445
Length of hospital stay	1.02	0.98–1.06	0.262
Baseline platelets	0.99	0.99–1.00	0.653
Baseline D-Dimer	1.32	1.12–1.55	0.0007
^a^Lung infiltrate (yes vs. *no*)	1.91	0.44–8.21	0.382

The logistic regression model is based on probability of having composite events

^a^Lung infiltrate defined as new consolidation diagnosed by chest x ray and or CT scan

#### Mortality

In total, 49 participants died, with an overall mortality rate of 7.7 %. The rate of mortality in the ICU group was 35.8 %, and that in the non-ICU group was 2 %. The participants were more likely to die if they were admitted to the ICU, older than 45 years, had a comorbidity, or had a composite event (Table 8).

**Table 8:** Risk Factors for Mortality in the Population

	*Hazard Ratio*	*95 % CI*	*p-value*
Age (≥ 45 years vs. *less than 45 years)*	5.4	1.6–18.2	0.006
BMI (≥ 30 vs. *less than 30*)	1.1	0.60–2.0	0.707
Admitted to (ICU vs. *non-ICU)*	5.0	2.3–10.8	< 0.0001
Cumulative comorbidities (≥ one vs. *0*)	1.6	0.8–3.3	0.035
Lung infiltrate (yes vs. *no*)	1.2	0.6–2.6	0.531
Composite event (yes vs. *no*)	2.3	1–5	0.035

## Discussion

In this multicenter study, we determined the thrombotic and bleeding complications of 636 COVID-19 patients. For VTE, all events except for one occurred in the ICU. The rate of VTE in the current study was much lower than that in previously published studies [10–14] but consistent with that in other studies [15–18]. In a multicenter study in the Netherlands (*n* = 184) [11] in which all patients were admitted to the ICU and received standardized doses of nadroparin and a dose escalation to 5700 IU BID at some point during admission in selected patients, the cumulative composite outcome of venous and arterial events was 49 % when adjusting for a competing risk of death. Of the PE group, 19 of 65 were subsegmental. Another retrospective study [12] included 198 patients (74 patients in the ICU). All patients in the ICU were given thromboprophylaxis at standard or double doses. After a median follow-up of 7 days (IQR, 3–13), 39 patients (20 %) had VTE. After adjusting for a competing risk of death, the cumulative incidence of VTE on day 21 was 59 % in the ICU group and 9.2 % in the ward group. Screening for VTE was performed in some patients at regular intervals. In a prospective study conducted in France [13], 150 ICU patients with ARDS were compared to patients admitted for non-COVID-19 ARDS. The primary outcomes were deep vein thrombosis, pulmonary embolism, myocardial infarction, mesenteric ischemia, lower limb ischemia, or cerebral ischemic attack. Propensity score matching was used, and it was determined that the COVID-19 ARDS patients had higher rates of thrombotic events than those with non-COVID-19 ARDS (11.7 % versus 2.1 %). In one of the largest studies that included 6513 patients (637 patients required mechanical ventilation), the rate of VTE within the hospitalized cohort was 3.1 %, and that in the subgroup of patients who required mechanical ventilation at any time during hospitalization was 7.2 % [15]. In another multicenter retrospective study with 400 admitted COVID-19 patients, the VTE (confirmed or presumed) rates were 6 %, 3.91 % and 10.4 % in all patients, noncritically ill patients and critically ill patients, respectively [16]. At the last ISTH meeting, a multicenter study that investigated the incidence of VTE and major bleeding in 3239 critically ill adults with COVID-19 was presented. The 14-day incidence of VTE was 6.3 %. It should be noted that 11.9 % of the patients received therapeutic anticoagulation, though the reason was not mentioned [18]. The low rate of VTE in the current study compared to that of earlier studies could be partially explained by the different thresholds for admission to the hospital or ICU. In our study, the mean patient age was lower than that in other studies. The definition of thrombotic outcome was not the same as that in other studies. For example, some studies included extracorporeal circuit thrombosis and microvascular thrombosis [13]. The follow-up period was variable, and different prophylaxis regimens were used (in the current study, more than 90 % of patients received pharmacological prophylaxis, and almost all patients in the ICU received prophylaxis). Additionally, there was no screening (Doppler US) for thrombotic complications, in contrast to other studies that reported a high rate [12]. Although we included suspected PE (not confirmed by imaging), some PE events could have been missed due to the difficulty associated with obtaining images in these patients, especially with the high mortality rate of 35 % in the ICU group, which could be explained by missed PE events. Regardless of all of these possible causes, we found that the VTE rate of patients admitted to the ICU (10.3 %) was similar to that described in other non-COVID-19 critically ill populations [20, 21]. What is also unusual is the very high PE-to-DVT ratio. Although PE may occur without DVT, it occurs in only 20 % of non-COVID-19 patients [22]. This high ratio may be due to in situ pulmonary artery thrombosis, a different pathophysiology of COVID-19. In terms of arterial complications, the current study had a high rate of arterial events in the ICU group (8.4 %; 95 % CI, 5.0–14.0). In the previously mentioned study [16], the rate of arterial thrombosis was 5.6 % (95 % CI, 2.4–10.7 %) in critically ill patients, which is lower than the rate in our study. Interestingly, most of the events in the current study were CVAs. Most CVAs occurred in ICU patients (6/10), but there were four events occurred in non-ICU patients. Despite that minor transient ischemic attack with normal scans could be missed, this rate is still high. There is no good explanation for this rate except that this disease is a true prothrombotic situation.

The diagnosis of MI was based on physician judgments, taking into consideration a combination of clinical, laboratory and other evidence and was not confirmed by a coronary angiogram. This may partially contribute to the high rate of arterial events as well.

Regarding bleeding complications, the overall bleeding rate of 1.7 % (95 % CI, 1.0–2.8) was similar to that in other COVID-19 studies [13, 18], but the rate of bleeding in the ICU group in the current study (9.4 %, 95 % CI, 5.7–15.1) was slightly higher than the rate of bleeding reported in a previous study 7.6 % (95 % CI, 3.9–13.3 %) [16]. Most of the bleeding events were nonmajor, and if we calculated the rate of major bleeding only, it was 3.7 % in the ICU group, which is similar to that of non-COVID-19 patients [23, 24]. We did not find correlation between the intensity of prophylactic regimen and bleeding as most of these bleeding events occurred with standard dose prophylactic regimens.

The difference in thrombotic outcomes in COVID-19 patients resulted in controversies in the guidelines of major societies for the prevention of these complications. The ISTH guidelines [25] suggested thromboprophylaxis with prophylactic-dose UFH or LMWH with the possibility of escalating to an intermediate‐dose for high-risk patients and a 50 % increase in the dose for obese patients. The CHEST guidelines [26] suggested the current standard-dose anticoagulant thromboprophylaxis over intensifying the dose to an intermediate or full treatment dose. In addition to an accurate estimate of the risk of thrombosis, there are areas requiring further investigation. There is limited evidence regarding the risk of bleeding in these patients. From the available evidence, the rate of major bleeding is variable and ranges from < 1 % in ward patients to 7.6 % in ICU patients [16, 18] (8.4 % in our study). This needs to be confirmed in well-designed prospective studies. A second issue is the case fatality rate of thrombotic and bleeding events, whether it is the same as for non-COVID-19 patients. This will support making a decision based on the risks and benefits of prophylaxis. There is also no definitive optimal method for assessing the risk for VTE. Would a risk assessment such as the Caprini, IMPROVE, or Padua model and others be able to risk stratify these patients? In terms of the diagnosis of VTE in COVID-19 patients, would a pretest probability using prediction models such as the Wells or Geneva model and biomarkers such as D-dimer be able to exclude VTE in these patients without the need for imaging studies (as it is known that most COVID-19 patients have a high D-dimer level)? Another unresolved issue is to determine the optimal dose of prophylactic anticoagulation and the duration of anticoagulation prophylaxis: should it be continued as an outpatient or not? In a large study of 1,877 patients [27], the rate of post discharge VTE was 4.8 per 1000 discharges over 42 days of follow-up, which is similar to the rate of discharge-associated VTE in non-COVID-19 medical admissions, with an odds ratio for post discharge HA-VTE associated with COVID-19 compared to non-COVID-19 of 1.6 (95 % CI, 0.77–3.1). As of 15 August 2020, more than 10 randomized clinical trials in which varying intensities of anticoagulation therapy were used and different outcomes, such as the thrombosis rate, mortality and other complications, such as ICU admission, were investigated were listed on clinicaltrials.gov. The evidence generated through these studies is expected to strengthen the evidence related to this special population.

The strengths of the current study are as follows. The first strength is the multicenter setting of the study: this study was conducted in four different hospitals, community-based and academic teaching hospitals, representing most of the population and increasing the probability of generalizability. The second, strength is the relatively large sample size. The third strength is at the end of data collection, all the participants were discharged or died, whereas in other studies, some patients remained in the hospital, which could have resulted in an underestimation of the outcome.

This study was limited by its retrospective nature. Although we included not only confirmed but also suspected cases of VTE, some may have been missed, as there was no uniform protocol to exclude VTE in any of the four centers. All outcomes were not adjudicated. In addition, the diagnosis of MI was based on the judgment of the physician, who took into consideration a combination of clinical, laboratory and other evidence, and was not confirmed by a coronary angiogram. Another limitation is the small number of ICU-admitted patients compared to non-ICU patients, which is probably secondary to a low threshold for the admission of COVID-19 patients with mild symptoms in these hospitals. Because of the change of pharmacological prophylaxis on frequent bases for same patient, we do not have data for the duration of pharmacological prophylaxis that have been prescribed for each patient. Due to the retrospective nature and because there was no uniform protocol for management of COVID-19, there could be missing values. For instance, baseline D-Dimer was not available for all patients.

## Conclusions

In this study, we found that the VTE rates in hospitalized patients with COVID-19 may not be higher than expected. In contrast to the risk of VTE, we found a high rate of arterial and bleeding complications in patients admitted to the ICU. An elevated D-dimer level at baseline could predict thrombotic complications in COVID-19 patients and may assist in the identification of these patients. Given the high rate of bleeding, the current study suggests that the intensification of anticoagulation therapy in COVID-19 patients beyond the standard of care be pursued with caution and is best evaluated in a randomized controlled study.

## Data Availability

The data used during the current study are available from the corresponding author on reasonable request.

## Abbreviations

ICU: Intensive care unit
VTE: Venous thromboembolism
COVID-19: Coronavirus disease 2019
RT-PCR: Reverse-transcriptase polymerase chain reaction
PE: Pulmonary embolism
DVT: Deep vein thrombosis
CVA: Cerebrovascular accident
MI: Myocardial infarction
DM: Diabetes mellites
ESRD: End stage renal disease
COPD: Chronic obstructive lung disease
GI: Gastrointestinal
CNS: Central nervous system
UFH: Unfractionated heparin
ISTH: International Society of Thrombosis and Haemostasis
CI: Confidence interval
